# The association between the sense of control and depression during the COVID-19 pandemic: a systematic review and meta-analysis

**DOI:** 10.3389/fpsyt.2024.1323306

**Published:** 2024-02-13

**Authors:** Rachel M. Msetfi, Diana E. Kornbrot, Yemaya J. Halbrook

**Affiliations:** ^1^ Maynooth University, Maynooth, Ireland; ^2^ Department of Psychology, Sport and Geography, University of Hertfordshire, Hatfield, United Kingdom

**Keywords:** public health restrictions, mental health, depression, sense of control, COVID, pandemic (COVID19)

## Abstract

**Introduction:**

High levels of depression and low sense of control have been reported during the COVID-19 pandemic. The removal of typical freedoms through public health restrictions may have played an important role. The aim of this review was to examine data collected during the pandemic and (1) estimate the strength of the association between sense of control and depression, (2) examine whether the different types of control measures affected the strength of the association, and (3) whether this changed as a function of pandemic indicators.

**Methods:**

We conducted a systematic review and meta-analysis of studies published in English between December 2019 and November 2022. A total of 993 articles were identified, of which 20 were included in the review and 16 in the meta-analysis after conducting a quality assessment using the standard NIH tool.

**Results:**

The control–depression association gave a bias-independent pooled effect size of *r* = .41, and grew stronger over the 130 weeks covered by this review but did not change as a function of local COVID incidence rates. Subgroup analyses showed that external and overall control were more strongly related to depression than internal control.

**Discussion:**

These findings emphasize that external factors are important to the sense of control and the importance of preserving the sense of control in situations where the removal of personal freedoms is necessary, such as public health emergencies.

## 1 Introduction

Sense of control is an important correlate of depression, with a lower sense of control predicting higher levels of depression (1–3). Low-control situations tend to induce cognitive, affective and behavioral changes that can result in depression (4). Given the removal of personal freedom during the COVID-19 pandemic, higher levels of depression are not unexpected (5). Therefore, the key aim of this study was to estimate the size of the association between sense of control and depression using data collected during the pandemic and to determine whether the type of control measured is a factor. A further key prediction tested is whether the strength of the control–depression association would change while the pandemic and as a function of pandemic indicators, such as incidence rates. This is because the sense of control would be predicted to change along with uncontrollable external factors, such as case numbers and changes in the levels of restrictions imposed by authorities. This review examined these questions.

### 1.1 Background

A large body of work has examined the sense of control and depression in the normal population, e.g (3)., and also in situations that might be considered uncontrollable, such as the case of aging populations (6), people with cancer (7), chronic illness (8), and life changing injuries (9). In all these examples, the relationship between sense of control and depression is evident and significant. People with a low sense of control tend to have higher levels of depression. Moreover, maintaining a sense of control, even if the overall outcome itself is uncontrollable, is key to coping with challenging situation (10).

It is important to note that different aspects of the sense of control have been studied. For example, Lachman and Weaver (3), like others, e.g (11)., distinguished between **internally focussed control**, sometimes labeled ‘mastery,’ which refers personal effectiveness in getting things done; and **externally focussed control**, labeled ‘perceived constraints,’ which refers to the external obstacles and factors external to the person which prevent them from reaching their goals. Interestingly, Lachman and Weaver (3) found that the relationship between externally focused control and depression (*r* range = |.24| to |.48|) was stronger than that between internally focused control and depression (*r* range = |.19| to |.27|). Similarly, Infurna and Mayer (12) noted that external control was more strongly related to mental health than internal control (13). Given the focus here on restrictions during the pandemic, we would expect that the lack of control with an external focus, such as the perception of large external obstacles blocking goals, would have a stronger predictive value in relation to depression than internal control during this time frame.

Therefore, the question to be answered here is whether the data collected by many researchers on mental health during the pandemic are consistent with this hypothesis. There is evidence of higher levels of depression. For example, a recent meta-analysis reported pre-COVID rates of 8.7% [95% Confidence Limits, CL: 6.2%, 11.5%], which increased to 18.3% during COVID [95% CL 13.5%–24.3%] in data collected up to July 2020 (14). Another meta-analysis (5) showed that depression levels were seven times higher than normal levels. Similarly data from the European COVID Survey, collected in November 2020 and April 2021, showed that the prevalence of ‘probable depression’ was very high at 26% (15). These studies, therefore, demonstrate sharp increases in depression levels during the pandemic, which is consistent with our predictions.

Furthermore, evidence also supports the suggestion that changes in the sense of control as a function of restrictions may have played a role in worsening depression. For example, several studies have shown that sense of control mediated or moderated distress during lockdown (16–18). For example, Gan et al. (18) found that, in China, the two-month impact of province-wide lockdown on psychological distress was moderated by personal control, such that the negative impact was greater in those with lower personal control. Senan et al. (19) found that a greater number of public health restrictions that were perceived as distressful predicted higher depression levels, but this effect was reduced when people had a stronger sense of control. Taken together, these studies provide evidence of a link between people’s subjective experience of the pandemic and their mental health, such that those with a lower sense of control fared worse in terms of higher levels of depression.

Current studies have several limitations in relation to the questions addressed in this study. For example, there is little evidence linking patterns of control and depression to external indicators of pandemic progression and severity, although there is evidence that distress levels change over the over the first few months. Gan et al.’s (18) study compared distress in lockdown and personal quarantine participants in China at two weeks and two months into the pandemic. Fancourt et al. (20) examined depression over the first 20 weeks of the lockdown in the United Kingdom and reported that initial increases in depression were alleviated over that time frame. We have not identified any other studies thus far that link psychological patterns to objective pandemic indicators, or directly examine the nature and changes in the sense of control and depression association. Given the speed at which data on mental health were collected and published during the pandemic, a systematic review is justified. In addition, the availability of open data on pandemic indicators makes it possible to examine mental health data alongside these indicators.

Thus, the aims of this study were to estimate the size of the association between sense of control and depression during the covid pandemic, identify the salient features of the same association, and check whether the effect size for the depression control association changes as a function of pandemic indicators, such as the local incidence rates of covid and the duration of the pandemic. To address these aims, we carried out a systematic review and meta-analysis with all the details described below.

## 2 Methods

The reporting of this systematic review and meta-analysis was guided by the 2020 PRISMA statement (21).

### 2.1 Search strategy

**Figure 1:**
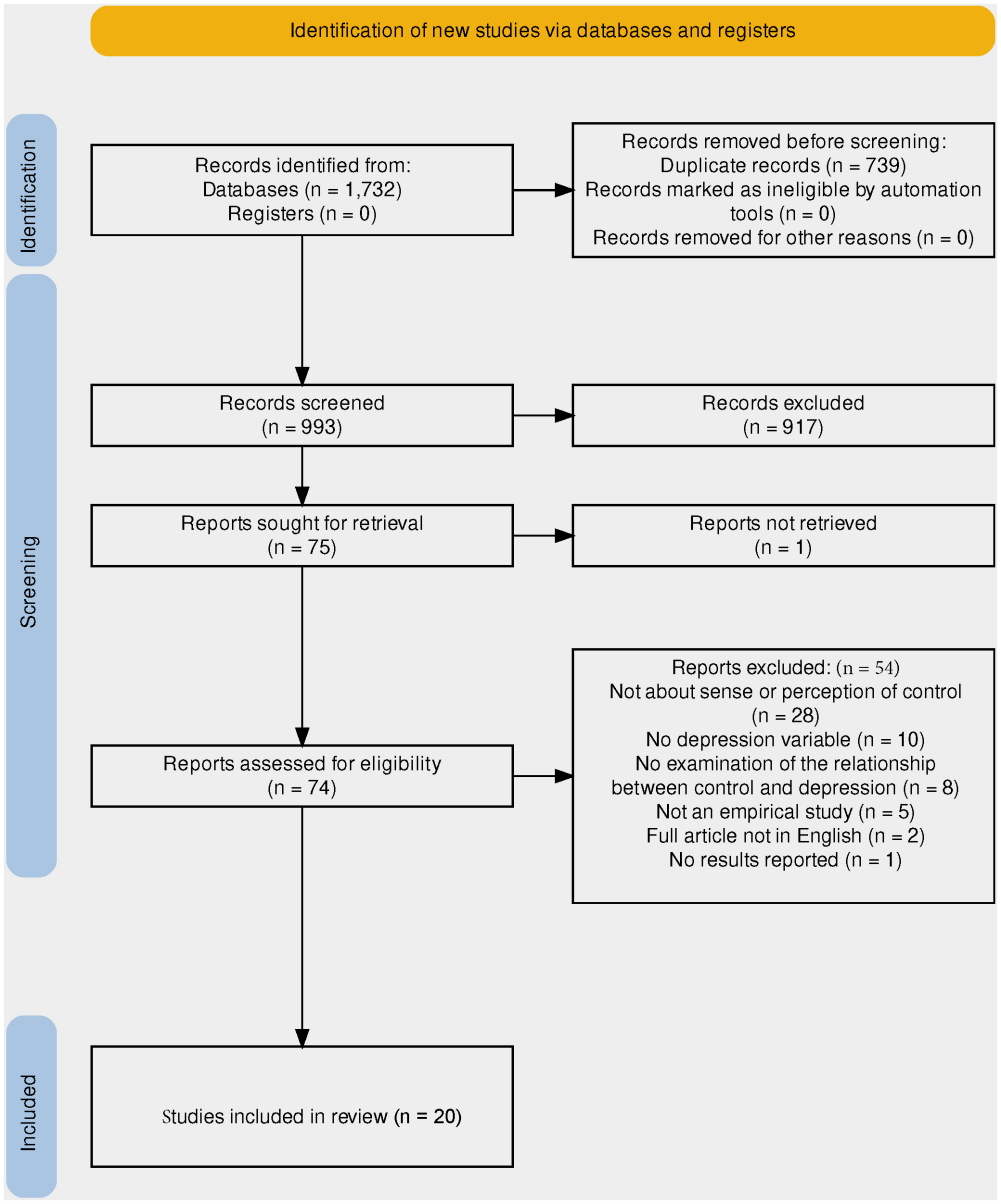
PRISMA flowchart (22) describing the identification and selection of studies for inclusion in the review. *Full article was not available.

Web of Science, SCOPUS, Embase, PubMed, PsycInfo, CINAHL complete, and EBSCO academic search complete databases were searched using the following keywords: ((covid) OR (covid-19) OR (pandemic) OR (SARS) OR (corona)) AND ((sense of control) OR (perception of control) OR (perceived control)) AND ((depression) OR (depressed) OR (depressive)) in titles, abstracts, and keywords in each database. The authors chose these search terms to address the research questions specified above. **Figure 1** shows a flow diagram of the search and selection processes. All papers identified in the searches were imported into Covidence software (23), which automatically removed duplicates. One author (YH) screened the titles and abstracts, while two authors (YH and RM) independently screened the full-text articles. The Covidence platform records agreements, disagreements, and resolutions between the reviewers.

### 2.2 Selection criteria

Studies were eligible for inclusion in this review if they reported the results of empirical investigations using quantitative measures of sense of control and depression, with the relationship between the two variables studied. The inclusion criteria were peer-reviewed journal articles ‘published in English’ after and including December 2019 and up until the final search date of 7 November 2022.

On the other hand, papers were excluded if they did not focus on sense of control, perception of control, or personal control, which are constructs that describe people’s views of themselves in relation to the environment. An example of an exclusion would be a study focusing on locus of control, which is considered to be a general orientation (24) or coping style (25), although it can be malleable to change (26). Studies were also excluded if they did not measure symptoms of depression or examine the relationship between sense of control and depression.

### 2.3 Quality assessment

The quality of studies was assessed using the National Heart, Lung, and Blood Institute of the National Institutes of Health (NIH) quality assessment tool for observational, cohort, and cross-sectional studies (27). This tool includes 14 criteria or questions that should be addressed; for example, “Was the research question or objective in this paper clearly stated?,” with outcomes being ‘yes,’ ‘no,’ or ‘other’ (including ‘cannot determine,’ ‘not applicable,’ ‘not reported’). In cases where an ‘not applicable’ outcome is not relevant to the quality rating, it does not count negatively to the rating. The evaluation was conducted independently by two of the authors (YH and RM), with any areas of initial disagreement discussed and a consensus reached. Initial inspection of quality evaluations indicated that bias was introduced into most studies due to low participant-to-population ratios (Q3, 19/20 studies) and use of cross-sectional designs (Qs 6, 7, and 10, 16/20 studies). More risky, in relation to the aims of the current review, were quality criteria related to the measurement of the predictor and outcome variables (Q9, sense of control, Q11, depression). Four studies used unusual measures of sense of control and one study used an unusual measure of depression. Other studies have used standard and well validated measurement tools. We weighted the predictor and outcome variable criteria most highly in our evaluation, as assessing the association between them was the key aim of the review, and unusual measurements would be predicted to introduce significant bias. Therefore, a weight of −2 was applied if there was non-compliance with criteria 9 and 11, with weight = −1 given to non-compliance with all other criteria. We then reviewed the overall scores and used them to inform but not to determine our overall evaluation. The classification process concluded with k = 3 studies classified at ‘good,’ k = 13 as ‘fair,’ and k = 4 as ‘poor.’ Note that studies classified as poor were classified as such in relation to the specific questions addressed in this review, which were not necessarily the focus of the original studies. Thus, our quality evaluation should not be interpreted as a general evaluation of the quality of these studies. The results of this quality assessment are presented in **Table 1**. The implications of the quality evaluation are described in the Results section.

**Table 1:** Quality assessment of studies included in the review using NIH guidelines for observational cohort and cross-sectional studies.

Study Authors	Study ID	Assessment Criteria														Score	Bias W	Final Rating
		1	2	3	4a	4b	5	6	7	8	9	10	11	12	13			
Alcover et al. (28)	#1	Yes	Yes	No	Yes	Yes	Yes	No	No	Yes	Yes	No	Yes	N/A	Yes	9/13	9/13	Fair
Crowe & Sarma (29)	#2	Yes	Yes	No	Yes	Yes	Yes	No	No	Yes	Yes	No	Yes	N/A	Yes	9/13	9/13	Fair
Curl & Wolf (30)	#3	Yes	Yes	No	Yes	Yes	Yes	No	No	Yes	**No**	No	Yes	N/A	Yes	8/13	7/13	Poor
Frazier et al. (31)	#4	Yes	Yes	CD	Yes	Yes	Yes	Yes	Yes	Yes	Yes	Yes	Yes	N/A	Yes	12/13	12/13	Good
Grace & VanHeuvelen (32)	#5	Yes	Yes	No	Yes	Yes	Yes	No	No	Yes	Yes	No	Yes	N/A	Yes	9/13	9/13	Fair
Hamm et al. (33)	#6	Yes	Yes	No	Yes	Yes	Yes	Yes	Yes	Yes	**No**	Yes	Yes	CD	Yes	11/14	10/14	Fair
Kondo et al. (34)	#7	Yes	Yes	CD	Yes	Yes	Yes	No	No	Yes	Yes	No	**No**	N/A	Yes	8/13	7/13	Poor
Mohammed et al. (35)	#8	Yes	Yes	No	Yes	Yes	Yes	No	No	Yes	Yes	No	Yes	N/A	Yes	9/13	9/13	Fair
Mohd Fauzi et al. (36)	#9	Yes	Yes	No	Yes	Yes	Yes	No	No	Yes	Yes	No	Yes	N/A	Yes	9/13	9/13	Fair
Msetfi et al. (37)	#10	Yes	Yes	No	Yes	Yes	Yes	Yes	Yes	Yes	Yes	Yes	Yes	Yes	No	12/14	12/14	Good
Precht et al. (38)	#11	Yes	Yes	No	Yes	Yes	Yes	No	No	Yes	Yes	No	Yes	N/A	Yes	9/13	9/13	Fair
Sahni et al. (39)	#12	Yes	Yes	No	Yes	Yes	Yes	No	No	Yes	**No**	No	Yes	N/A	Yes	8/13	7/13	Poor
Senan et al. (19)	#13	Yes	Yes	No	Yes	Yes	Yes	No	No	Yes	Yes	No	Yes	N/A	Yes	9/13	9/13	Fair
Shinan-Altman & Levkovich (40)	#14	Yes	Yes	No	Yes	Yes	Yes	No	No	Yes	Yes	No	Yes	N/A	Yes	9/13	9/13	Fair
Skapinakis et al. (41)	#15	Yes	Yes	No	Yes	Yes	Yes	No	No	Yes	Yes	No	Yes	N/A	Yes	9/13	9/13	Fair
Sugawara et al. (42)	#16	Yes	Yes	No	Yes	Yes	Yes	No	No	Yes	Yes	No	Yes	N/A	Yes	9/13	9/13	Fair
Van Mulukom et al. (43)	#17	Yes	Yes	No	Yes	Yes	Yes	No	No	Yes	**No**	No	Yes	N/A	Yes	8/13	7/13	Poor
Wanberg et al. (44)	#18	Yes	Yes	No	Yes	Yes	Yes	Yes	Yes	Yes	Yes	Yes	Yes	Yes	Yes	13/14	13/14	Good
Wierenga et al. (45)	#19	Yes	Yes	No	Yes	Yes	Yes	No	No	Yes	Yes	No	Yes	N/A	Yes	9/13	9/13	Fair
Xiong et al. (17)	#20	Yes	Yes	CD	Yes	Yes	Yes	No	No	Yes	Yes	No	Yes	N/A	Yes	9/13	9/13	Fair

NB: Yes, green shading; No, red shading; Not reported, yellow shading; Cannot determine, CD, gray shading; Not applicable, N/A, blue shading. Score ratios do not include unapplicable criteria. Criteria were scores <55% = Poor, 55 to 75% = Fair, and 75%+ = Good; Score = total Yes/Total relevant criteria. Bias W indicates total score adjusted for criteria weighted more highly (*2) for the purposes of this review (Q9 and 11).

### 2.4 Data acquisition and coding

Data were extracted from each paper either through Supplementary Information or raw data supplied by the authors. These data included the effect and sample sizes, as well as the start and end dates of data collection. Overall, 38 effect sizes were derived from 20 manuscripts. In addition, data on global 14-day incidence rates per 100,000 COVID-19 cases per country during the pandemic were retrieved from the European Centre for Disease Prevention and Control (46). These data were reported weekly throughout the pandemic, with week 1 representing the first week of January 2020. The dataset ceased to be updated on 1 November 2022.

The ECDC data were matched to the data collection time frame of the retrieved studies per week, from weeks 1 to 128 (where weeks 53 to 104 represent 2021). Where mental health data collection commenced prior to 1 January 2020, or the ECDC reported NA in relation to cases, 0 cases were assumed. Data collection weeks prior to 2020 were given negative values (i.e., −1, −2, etc.) for these analyses.

Given our aim to analyze mental health data alongside COVID data and the time frame of the pandemic for a given location, where data for multiple countries were summarized in the publication, these data are reported here per country, where sample size per country permitted. We also included the continent classification obtained from the ECDC data in the analysis. Where necessary, the corresponding authors were contacted to clarify the data collection time windows and locations, and in some cases, to obtain the raw data so that the relevant values could be recalculated. Values were calculated from the raw data supplied to us, as indicated in the data summary.

### 2.5 Target variables

The main target variable was the absolute value of the effect size *r* derived from the simple correlation between sense of control and depression. Where mean differences were available, Cohen’s d was calculated and converted to *r*. If β values were provided from multiple regression analyses, the simple *r* value was used if reported, obtained from Supplementary Data, or recalculated from the raw data; otherwise, the β was converted to r. The standard error of *r* and weight of each case were calculated for each of the 38 effect sizes found in the 20 included articles. The *r* values were then transformed using Fisher’s *r*-to-*Z* transformation, (ES_Z_). All statistical analyses were performed using ES_Z_ as the target variable.

The key predictor variables in the data set were data collection start week, study duration, COVID incidence rates during start week and end week (indices of pandemic severity), study continent, and type of control (categorized as “internal” referring to mastery or the ‘I’ focussed control, “external” referring to external constraints or external forces which affect the individual’s control, or “overall” control, and a general measure which encompasses both of these factors and other aspects of control.

### 2.6 Analyses

The meta-analyses were conducted using the RStudio (Version 2022.12.0 + 353). The Metafor package (47) was used to conduct three-level meta-analyses so that multiple effect sizes from each study could be used where available, thus accounting for measure dependence by nesting each measurement within the study. Nested three- and two-level models were examined, with the nested model providing a better fit, AIC and BIC_Nested_ <AIC and BIC_2 level_, the likelihood ratio test was significant (χ^2^ = 32.91, p <.00001). Therefore, nested models were used in this study. Meta-analyses were conducted on Fisher z transformed r-values to avoid bias.

## 3 Results

### 3.1 Study characteristics

The search identified 1,732 manuscripts, of which 739 were duplicates. The remaining 993 titles and abstracts were screened using 75 full-text articles that were assessed for eligibility. After applying the exclusion criteria, 20 manuscripts remained with a total of 27,685 participants. The countries covered were Asia (India, China, Korea, Malaysia, Japan), the Middle East (Egypt, Israel, Saudi Arabia), Americas (Brazil, USA, Canada), Europe (Croatia, Finland, France, Greece, Germany, Ireland, Italy, Portugal, Spain, UK), and Australia.

All 20 studies reviewed used a cross-sectional survey design approach. Of these, two were longitudinal variables measured at multiple time points. Two other studies compared data collected prior to the pandemic with data collected to determine if the levels of sense of control and depression changed. The remaining 16 studies examined data collected during a specified timeframe of survey distribution, which varied in duration from one to 49 weeks, with a median data collection time window of 4 weeks (*M* = 8.38, *SE* = 1.83). Further information is provided in **Table 2**, including measures of depression and controls used, the size and nature of samples, the location and timing of the study, and key findings.

**Table 2:** Characteristics and findings for all 20 studies included in the review.

Author and Study ID #	Location; date range	Sample	Age range	Design	Findings	Statistics/ES
Alcover, et al. (28) #1	Spain; 13/04/20 to 20/04/20	Adults: *N* = 421	17–89, M = 45.38	Cross-sectional online survey study utilizing both a correlational analysis for the relationship between depression and control as well as a Kruskal–Wallis analysis comparing categorical low, medium, and high levels of control on depression.	Those with higher perceived personal control reported lower levels of depression than those with low perceived control. This was demonstrated through both correlational analyses as well as a categorical high, medium, and low control variable.	Relationship between depression and perceived control: *r* = −.36, *p* <.001, medium ES Differences between three levels of perceived control on depression: Eta-squared calculated from Kruskal-Wallis H-statistic: η^2^ = .118, medium ES
*Crowe and Sarma (29) #2	Ireland; 10/01/21 to31/01/21	Adult pregnant women; *N* = 761	18+	Cross-sectional online survey study utilizing t-tests and ANOVAs to investigate the relationship between demographic factors and psychological distress. A series of hierarchical linear regressions were conducted to measure if lower levels of perceived control was associated with psychological distress. Lastly, moderation model using Hayes PROCESS was conducted to test if the relationship between COVID-19 related pregnancy concern and psychological distress is moderated by perceived control.	Those with a lower sense of perceived control had significantly higher levels of psychological distress, a combination variable of depression, anxiety, and prenatal distress. This was demonstrated through both correlational and regression analyses. However, sense of control did not moderate the relationship between COVID-19 related pregnancy concerns and psychological distress.	Relationship between perceived control and psychological distress: *r* = −.56, *p* <.01, large ES Regression of perceived control on psychological distress: β = −.39, *p* <.001, medium ES Perceived control did not moderate the relationship between COVID-19 related pregnancy concerns and psychological distress.
*Curl and Wolf (30) #3	USA, excluding Hawaii and Alaska; 11/6/20 to 15/05/21	Adults >50 years: *N* = 2,145	51–99, M = 69.06	Structural equation modeling was conducted to determine if either measure of control predicted levels of depression. Data is continuously being collected during the pandemic and the data gathered between March 2020 and May 2021 is what is utilized in this study.	Structural equation modeling determined that feeling control over both health and social life during the pandemic predicted lower levels of depression than feeling less control.	In relation to depression: Control over health: β = −0.14, *p* <.05; Small ES Control over social life: β = −0.19, *p* <.05; Small ES
Frazier, et al. (31) #4	USA; April 2020 compared to Spring 2017, where 2020 collection was 7/04/20 to 12/04/20	Students: Spring 2017: *n* = 362; April 2020: *n* = 312	College aged; M= 21.11, 2017; M = 19.98, 2020	Comparisons were made between two different undergraduate samples from 2017 and 2020, pre vs during pandemic controlling for differences between samples. MANCOVAs were conducted to compare samples while controlling for demographics that differed across the two samples. Correlational analyses were used to examine the relationships in the 2020 sample.	Moderate symptoms of depression increased from 28% in 2017 to 49% in April 2020. On the other hand, perceived present control decreased between the two timepoints with a significant relationship to depression such that the lower perceived present control, the higher the depression.	Comparisons between the 2017 and 2020 groups: Depression: Differences between mean scores (without controlling for demographics): Glass *d* = .53, *t*(597) = 7.31, *p* <.001, medium ES. Univariate follow-up from MANCOVA on depression (controlling for demographics): *F*(1, 631) = 40.18, *p* <.001, partial η^2^ = .06, medium ES Perceived present control (controlling for demographics): F(1, 627) = 9.29, *p* = .002, partial η^2^ = .02, small ES Significant negative correlation between perceived present control and depression in 2020 sample: *r* = −.45, *p* <.001, medium ES.
Grace and VanHeuvelen (32) #5	USA; 08/07/20 to 13/10/20	Adults: *N* = 2,000 (of which those who experienced bereavement: *n* = 184)	18–65+	Cross-sectional survey study where ordinary least squares (OLS) regression models examining how COVID-19 bereavement associates with depression. OLS exploring associations of mastery with depression. OLS predicting depression between all demographic variables.	Those who experience bereavement reported higher levels of depression and lower levels of mastery than those who did not. For all participants, those who reported a greater mastery reported less symptoms of depression.	Bereavement on depression: β = .51, *p* <.001, large ES; Mastery on depression: β = −0.44, *p* <.001, medium ES; Bereavement on mastery: β = −0.15, *p* <.05, small ES
Hamm, et al. (33) #6	USA; T1 16/04/20 T2 01/05/20 T3 17/06/20	Adults: *N* = 292	18–80, M = 45	Longitudinal two-month online survey with wave utilising the same questionnaire to compare scores across timepoints. Variables were averaged across time-points for overall relationships using correlations. Multilevel growth models were used to examine changes across the two-months.	Those with high perceived control over their goals had more adaptive levels of depression but did not predict changes in depression over time. Further, for those with low perceived control, having goal reengagement predicted lower depressive symptoms, indicating that while higher control may elicit lower depression, goal reengagement may buffer this relationship. Depression levels also declined overall across the two months.	Depression declined over the two-months, y = −.05, *SE* = .011, *p* <.001. Averaging depression and control across waves, the correlational relationship: *r* = −.31, *p* <.05, medium ES (available in Supplementary Data).
*Kondo, et al. (34) #7	Japan; 04/11/20 to 24/05/21 (#7a); USA; 01/11/20 to 25/05/21 (#7b)	Adults Japan: *n* = 739; USA: *n* = 139	Japan, M = 24.3; USA, M = 31.3	Cross-sectional online survey. T-tests were performed to compare overall perceived control between countries with chi-square tests conducted to compare two categories of mental health effect. Spearman’s correlation coefficient was used to examine the relationship between perceived control and mental health effect. Variables that were significantly related to mental health effects were included in a hierarchical multiple logistic regression with perceived control being entered in the fifth level of six total levels for nursing students only.	Feelings or symptoms of depression were higher and perceived control was lower in Japanese students when compared to American students. Higher mental health effects were related to lower perceived control for Japanese students, but not for American students. However, after adjusting for perceived control, there was no difference between countries for stress and/or depression. This indicates that if perceived control were similar, so would be depression between countries.	Differences between Japan and USA for perceived control: *t* = 8.86, *p* <.001, Cohen’s *d* = .60, medium ES. Differences between Japan and USA for mental health effects: χ2 = 16.6, *p* <.001, φ = .18, small ES. Correlation for both samples of perceived control and mental health effect: *r* = −.135, *p* <.001, small ES Hierarchical linear regression for nursing students of perceived control on mental health effect: Odds ratio = .97, CI [.94,.99], small ES
Mohammed, et al. (35) #8	Egypt; 01/12/19 to 15/03/20	Students: *N* = 766	18+, M = 21.27	A cross-sectional survey was conducted, though it does not specify if it was online or if the recruitment and survey were in person. A t-test was used to compare means of sense of control between depressed and non-depressed students. Chi-square and Fisher’s exact tests were also used to compare proportion between groups. Lastly, logistic regression models to applied to identify predictors of depression.	Sense of control significant predictor of depression.	Sense of control values were significantly higher in students with depressive symptoms (*M* = 2.89, *SD* = 0.8) than those without depressive symptoms (*M* = 2.27, *SD* = 0.64), *p* <.05, Mean difference converted to Cohen’s *d* = 0.846. Logistic regression (univariate): Odds ratio = 3.453, CI 95% [2.722, 4.381], *p* <.001, medium ES Multivariate: Odds ratio = 2.323, CI 95% [1.763, 3.060], *p* <.001, ES = 1.283, small ES.
Mohd Fauzi, et al. (36) #9	Malaysia; 01/05/20 to 31/05/20;	Health care professionals: *N* = 1,050	24–59, M = 33.08	Online cross-sectional survey. Multiple linear regressions and correlational analyses were conducted to determine the associations between variables while controlling for sociodemographic factors such as gender and age. No specific demographic comparisons were made.	There was a negative relationship between depression and mastery as well as depression and control over leisure time. Additionally, mental, physical, temporal, and emotional demand of work all relate higher levels of depression and lower levels of control. Although participants in this sample did not suffer from depression overall, this negative relationship indicates that perceived control could have served as a protective factor against depressive symptoms.	When including all four work demand constructs, detachment, control, relaxation, and mastery in the multiple linear regression, the overall *R* ^2^ = .30, large ES. Multiple linear regression: Depression and control: Adj. *b* = −.08, CI 95% [−.15, -−02], *p* = .01, small ES Depression and mastery: Adj. *b* = −.11, CI 95% [−.16, −.06], *p* <.001, small ES Correlational analysis: Depression and control: *r* = −.293, *p* <.001, small ES Depression and mastery: *r* = −.308, *p* <.001, medium ES Control and mastery: *r* = .395, *p* <.001, medium ES
*Msetfi, et al. (37) #10	Republic of Ireland; T1 07/01/22 to 22/02/22 #10a; T2 07/05/22 to 23/06/22 #10b	Adults: T1 *N* = 314 T2 *n* = 47	18–76, M = 27.79 (T1); 19–51, M = 29.57 (T2)	Online cross-sectional survey. T-tests were first conducted to compare the low and high BDI status groups on the 4 predictor variables. A logistic regression was conducted to examine the effect of perceived control on categorial low/high BDI values for both Time 1 and Time 2 values. ANOVAs were additionally conducted to evaluate changes in values over the two time-points.	T1 high BDIs < control low BDIs, perceived constraints significantly predicted BDI category;	Mastery and BDI: *t* = 7.61, *p* <.001, Cohen’s *d* = .86, large ES Perceived constraints and BDI: *t* = 12.48, *p* <.001, Cohen’s *d* = 1.41, large ES
*Precht, et al. (38) #11	Germany; 12/10/20 to 30/11/20	Students: *N* = 568	16–66, M = 19.90	Online cross-sectional survey. Correlational analyses were conducted to measure relationships between variables as well as mediations including physical activity as an independent variable, sense of control as a mediation and depression as the dependent variable. Age and gender were included as covariates.	Sense of control significant predictor of depression; Sense of control was demonstrated to significantly mediate the relationship between physical activity and depression.	Correlational relationship between control and depression: *r* = .522, *p* <.001, large ES Mediation between physical activity and depression with sense of control as a mediator: Indirect effect: *b* = −.418, *SE* = .108, CI [−.632, −.210]
Sahni, et al. (39) #12	India; 26/04/20 to 08/06/20	Adults: *N* = 643; yoga practitioners: *n* = 384; non-yoga practitioners: *n* = 259; spiritual practitioners: *n* = 113	18–72, M = 28.12	Online cross-sectional survey. Correlational analyses were conducted to examine relationships between variables. MANOVAs were then conducted to examine differences between sample groups on the dependent variables.	Those who practiced yoga reported significantly higher levels of personal control, perception of preventative control, and lower levels of depression than those who did not practice yoga, whether they were spiritual practitioners or not. Supplementary Data demonstrated a significant negative relationship between depression and sense of control.	Relationship between personal control over illness and depression: *r* = −.135, *p* <.05, small ES. Relationship between control over treatment and depression: *r* = −.017, *p* <.05, small ES Practitioner group on personal control: partial η^2^ = .051, *p* <.001, small ES Practitioner group on depression: partial η^2^ = .058, *p* <.001, small ES
Senan, et al. (19)* #13	Saudi Arabia; 16/11/20 to 26/12/20	Adults: *N* = 641; *n* high BDI = 295, *n* low BDI = 346.	18–65+	Online cross-sectional survey. Binary logistic regression was utilized with depression categorized as high and low with all predictor variables and interactions between predictor variables being entered into the analysis.	Public health restrictions significantly predicted depression levels, this relationship was reduced by high levels of sense of control. More specifically, for those with a high or very high sense of control, higher restriction numbers did not significantly increase the likelihood of depressive symptoms whereas the likelihood of depressive symptoms increased by 32.7% with increased restriction numbers for those with low sense of control.	ESs on depression: Constraints: *B* = −.50, *W* = .10, large ES Mastery: *B* = −.71 *W* = .30, large ES Mastery x restrictions × impact: *B* = .01, *W* = .12, small ES Constraints x restrictions × impact: *B* = −.19, *W* = .13, small ES
Shinan-Altman and Levkovich (40) #14	Israel; 01/01/21 to 02/02/21	Teachers: *N* = 208	24–65, M = 43.4	Online cross-sectional survey. Correlational analyses were conducted to examine relationships between variables. A path analysis was conducted using AMOS to examine model fit of the variables.	Higher levels of sense of control are negatively associated with levels of depression such that the higher the sense of control, the lower the depression. Further, perceived stress mediated the relationship between sense of control and depression indicating that a higher sense of control can elicit lower perceived stress which, in turn, elicits lower levels of depression.	Correlational relationship between sense of control and depression: *r* = -.44, *p* <.001, small ES Indirect effect between sense of control and depression with stress as a mediator: *B* = -.13, CI [-.20, -.07], *p* <.001
Skapinakis, et al. (41) #15	Greece; 08/04/20 to 12/04/20	Adults: *N* = 3,379	18+, M = 42	Online cross-sectional survey. A binary logistic regression with depression as a categorical variable was conducted to examine various independent variables on levels of depression.	Those with higher senses of personal control and treatment control were less likely to experience high levels of depression.	High personal control over illness on depression: Odds ratio = .79, CI 95% [.65,.96], *p* = .002, small ES High control over treatment on depression: Odds ratio = .62, CI 95% [.49,.79], *p* <.001, small ES. *ES from multivariable analysis
Sugawara, et al. (42) #16	Japan #16a, Malaysia #16b, China #16c, USA #16d; all locations 14/10/20 to 02/11/20.	Adults: *N* = 1,583 16a, *n* = 322 16b, *n* = 423 16c, *n* = 505 16d, *n* = 333	19–82, M = 32.22	Online cross-sectional survey. Correlational analyses were conducted to explore relationships between variables. Additional hierarchical multiple linear regressions with four steps were conducted to examine the interaction between fear of COVID-19 and the impact of resilience factors on mental distress. Step 1: mental distress as dependent variable and demographics as control variables. Step 2: Fear of COVID-19 Step 3: sense of control, ego-resilience, grit, and self-compassion Step 4: Interaction between fear of COVID-19 and each of the four resilience factors.	Sense of control was negatively correlated with mental distress, here measured as combined depression and anxiety. This was further demonstrated using a hierarchical linear regression which showed sense of control was significantly associated with mental distress for all countries. There was also a significant interaction effect between sense of control and fear of COVID-19 on mental distress for the Chinese sample and total dataset only, but not for Japan, US, or Malaysia individually. This indicates that a low sense of control leads to a higher chance of the fear of getting infected affecting mental distress in a Chinese population as well as overall across countries, but perhaps not so in the US, Japan, or Malaysia.	Relationship between mental distress and sense of control: *r* = −.53, *p* <.01, large ES *R* ^2^ = .52 for total sample when fourth step is added, large ES. Sense of control on mental distress at third step: β = −0.36, *p* <.01, medium ES.
*Van Mulukom, et al. (43) #17	Global survey—79 countries included; 28 March–24 April 2020;	Adults: *N* = 8,229; 17a to 17i Australia *n =* 683 Brazil *n =* 884 Croatia *n =* 209 Finland *n =* 219 France *n =* 237 Italy *n =* 1,029 Portugal *n =* 367 UK *n =* 1,082 USA *n =* 2,167	18–88, M = 38.3	Online cross-sectional survey. R was used for linear regressions and structural equation modeling to examine the overall hypothesized model.	A low sense of control predicted symptoms of depression. Sense of control itself was predicted by maladaptive coping (negatively) and adaptive coping (positively), as well as frequency of communication about COVID-19 (negatively), and government actions and perceived knowledge (positively). These relationships indicate that coping and communication may help elicit a stronger sense of control which would, in turn, help protect against feelings of depression.	In the model, sense of control on depression: β = −0.31, *p* <.001, medium ES.
Wanberg, et al. (44) #18	USA; T1 April–June, 2019 T2 16/04/20 to 19/04/20	Adults: *N* = 1,143	30–80	Online cross-sectional study given at two timepoints to the same participants. One-sample and paired sample t-tests were used to examine pre and during pandemic scores. Structural equation modeling was used to examine education and income on various mediators, sense of control included, and this in turn on depression. Latent change score modeling to compare increases in depression between socioeconomic groups.	Depression significantly increased between the two timepoints for the same participants at each timepoint. Additionally, lower income significantly predicted higher levels of depression which was further mediated by a sense of control. More simply, a lower income leads to a lower sense of control which ultimately leads to higher levels of depression. These findings indicate that those of a lower SES may need targeted sense of control interventions.	Relationship between control and depression pre-pandemic: *r* = −.17, *p* <.01, small ES During pandemic: *r* = −.47, *p* <.01, medium ES SEM control on depression during pandemic: β = −0.99, *SE* = .37 *p* <.01, large ES
Wierenga, et al. (45) #19	USA; 23/03/20 to 02/06/20	Adults *N =* 1,380	18–89	Online cross-sectional survey. Correlational analyses were conducted to examine relationships between variables. As all participants reported being mildly to moderately depressed, there was no non-depressed participants for comparison purposes.	All participants reported being mild to moderately depressed overall with moderate feelings of personal control and treatment control. However, there were no significant relationships between these two constructs of control and depression indicating that for this already depressed sample, feelings of personal control and treatment control were not associated to feelings of depression. Additional non-depressed participants would be needed for more robust conclusions.	Relationship between personal control and depression: *r* = −.03, *ns* Treatment control and depression: *r* = −.01, *ns*
Xiong, et al. (17) #20	China; 20/02/20 to 20/03/20	*N* = 563 Medical students *n = 382* Non-medical students *n* - 181	M = 21.52	Online cross-sectional survey. Correlational analyses were conducted to explore the relationships between variables. Independent two-sample t-tests were conducted to compare continuous variables with chi-square analyses to compare categorial variables. Linear regressions were further conducted to explore possible prediction effects.	Non-medical students in China experienced overall higher symptoms of depression than medical students. However, total score for sense of control, perceived constraints, and perceived mastery were significantly negatively related to depression for both groups, indicating that having a higher sense of control would be helpful for all students, regardless of discipline. For the regression analyses, perceived constraints, but not perceived mastery, predicted depression for both medical and non-medical students.	Relationships to depression in medical students: Overall control: *r* = −.46, *p* <.001, medium ES Constraints: *r* = −.30, *p* <.001, medium ES Mastery: *r* = −.45, *p* <.001, medium ES In non-medical students: Overall control: *r* = −.50, *p* <.001, large ES Constraints: *r* = −.34, *p* <.001, medium ES Mastery: *r* = −.48, *p* <.001, medium ES

*Indicates studies where authors have supplied further information including dates of data collection and raw data.

All studies, except one (45), reported a significant relationship between sense of control and depression. When comparisons were reported between data collected prior to and during the pandemic, depression levels were higher and sense of control levels were lower in 2017 than in 202 (31), and those studies that compared depression levels at several points during the pandemic reported that levels decreased over time (33, 37).

### 3.2 Quantitative analyses

A summary of these data is presented in **Table 3**. The effect sizes varied from *r* = .006 to.57. Of the 20 studies, 14 studies reported simple correlations, and five reported other effect size values or descriptive statistics, which were converted to *r* and then Fishers z transformed. The authors of one study provided raw data, and the simple correlation between depression and one of the control items was re-calculated by the current authors. Overall, 38 effect sizes were obtained.

**Table 3:** Summary findings and data details.

Study ID #	Location	Date range	Sample size	Control measure	Control Type	Depression measure	Direction	ES	ES *r*
1 (28)	Spain	13/04/20 to 20/04/20	421	PPC^56^	Internal	PHQ-9	−	Reported	.36
2* (29)	Ireland	10/01/21 to31/01/21	761	SOC	Overall	Psychological Distress	−	Reported	.56
3*^1^ (30)	USA	11/6/20 to 15/05/21	2,145	Health and Social control	Internal	CES-D	−	Converted	.14
4 (31)	USA	April 2020 compared to Spring 2017, where 2020 collection was 7/04/20 to 12/04/20	Spring 2017: *n* = 362; April 2020: *n* = 312	PCSE	Internal	DASS-21	−	Reported	.45
5 (32)	USA	08/07/20 to 13/10/29	2,000	PMS	Internal	CES-D		Reported	.44
6 (33)	USA	T1 16/04/20 T2 01/05/20 T3 17/06/20	292	Single item question (Mastery)	Internal	CES-D10	−	Reported	.31
7*^1^ (34)	Japan USA	04/11/20 to 24/05/21 (#7a); USA; 01/11/20 to 25/05/21 (#7b)	Adults Japan: *n* = 739; USA: *n* = 139	BGHS	Overall	Two questions	−	Reported	Japan.11 USA.14
8 (35)	Egypt	01/12/19 to 15/03/20	Students: *N* = 766	SOC	Overall	PHQ-9	+	Converted from mean differences	.39
9 (36)	Malaysia	01/05/20 to 31/05/20;	Health care professionals: *N* = 1,050	REQ-mod	Overall	DASS	−	Reported	.29
10* (37)	Ireland	T1 07/01/22 to 22/02/22; T2 07/05/22 to 23/06/22	T1 *N* = 314 T2 *n* = 47	SOC	Internal/External	BDI	−	Converted from mean differences	T1.396 T2.449
11* (38)	Germany	12/10/20 to 30/11/20	568	SOC-2	External	DASS-21	+	Reported	.522
12^1^ (39)	India	26/04/20 to 08/06/20	643	BIPQ	External	DASS-9	−	Reported	.440
13* (19)	Saudi Arabia	16/11/20 to 26/12/20	*n* high BDI = 295, *n* low BDI =346	SOC	Internal/External	BDI	−	Converted from mean differences	.309
14 (40)	Israel	01/01/21 to 02/02/21	208	7-item scale	Internal	CES-D10	−	Reported	.440
15 (41)	Greece	08/04/20 to 12/04/20	*N* = 3,379	IPQ-R	Internal	PHQ-9	−	Converted from multi variable analysis	.006 (MVR)
16 (42)	Japan Malaysia China USA	All locations 14/10/20 to 02/11/20.	*N* = 1,583 16a, *n* = 322 16b, *n* = 423 16c, *n* = 505 16d, *n* = 333	SOC	Overall	DASS-21	−	Reported	16a.55 16b.57 16c.57 16d.33
17*^1^ (43)	Global survey—79 countries included;	28 March–24April 2020;	*N* = 8,229; Australia *n =* 683 Brazil *n =* 884 Croatia *n =* 209 Finland *n =* 219 France *n=*237 Italy *n=*1029 Portugal *n=*367 UK *n=*1082 USA *n=*2167	Actor Director Questions^1^	Internal	HADS	−	Calculated from raw data	17 0.43 17a 0.497 17b 0.416 17c 0.427 17d 0.400 17e 0.351 17f 0.365 17g 0.368 17h 0.471 17i 0.434
18 (44)	USA	T1 April–June, 2019 T2 16/04/20 to 19/04/20	1,143	SOC	Internal	PHQ-8	−	Reported	.54
19 (45)	USA	23/03/20 to 02/06/20	1,380	BIPQ-covid	Internal	PHQ-8	ns	Reported	.03
20 (17)	China	20/02/20 to 20/03/20	563	SOC	Overall	DASS-21	−	Reported	.46

*Indicates studies where authors have supplied further information including dates of data collection and raw data. ^1^These studies were not included in quantitative analyses designed to answer key research questions.

We wanted to check whether studies classified as ‘poor’ should be included in further quantitative analyses. Three-level meta-analysis models were fitted which estimated the overall *ES_Z_* across all effect sizes or only across those derived from studies classified as ‘fair’ or ‘good.’ Quality classification was included in each model as a categorical moderator. With all studies included in the model, the effect of quality approached reliability (*Q_M_* (2) = 5.66, *p* = .0591). However, with k = 4 ‘poor’ studies removed from the model, quality did not have a reliable effect, *Q_M_* (1) = 1.16, *p* = .2823. Therefore, we conducted the remaining analyses with only 16 studies that were categorized as fair or good with 24 nested observations. We were able to use this data to answer the three questions posed below.

#### Q1. What was the pooled size of the association between sense of control and depression during the covid pandemic?

**Figure 2:**
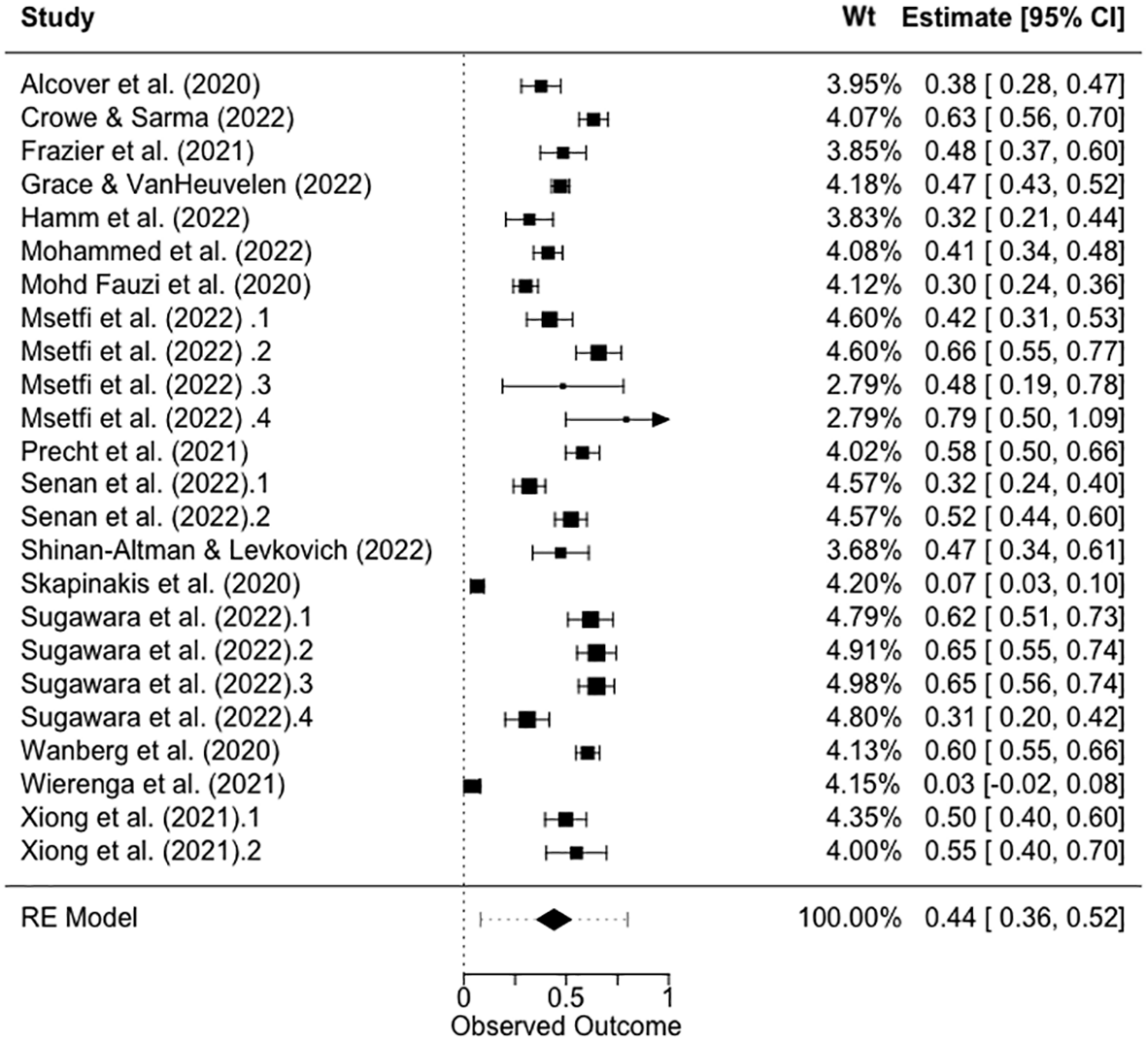
Forest plot showing the *ES_Z_* and pooled *ES_Z_* for the relationship between sense of control and depression. NB: 95% confidence limits are shown in square brackets.

The overall results are shown as a forest plot in **Figure 2**. The pooled effect size was significant, *ES_Z_* = .44 [95% CL:.36,.52], *Z* = 10.40, *p* <.0001, with significant heterogeneity, *Q_E_* (23) = 798.90, *p* < .001. This *ES_Z_* converts to *r* = .41, which is a medium to large effect size, using Cohen’s (48) conventions.

#### Q2. What are the salient features of sense of control and depression association during the pandemic?

**Figure 3:**
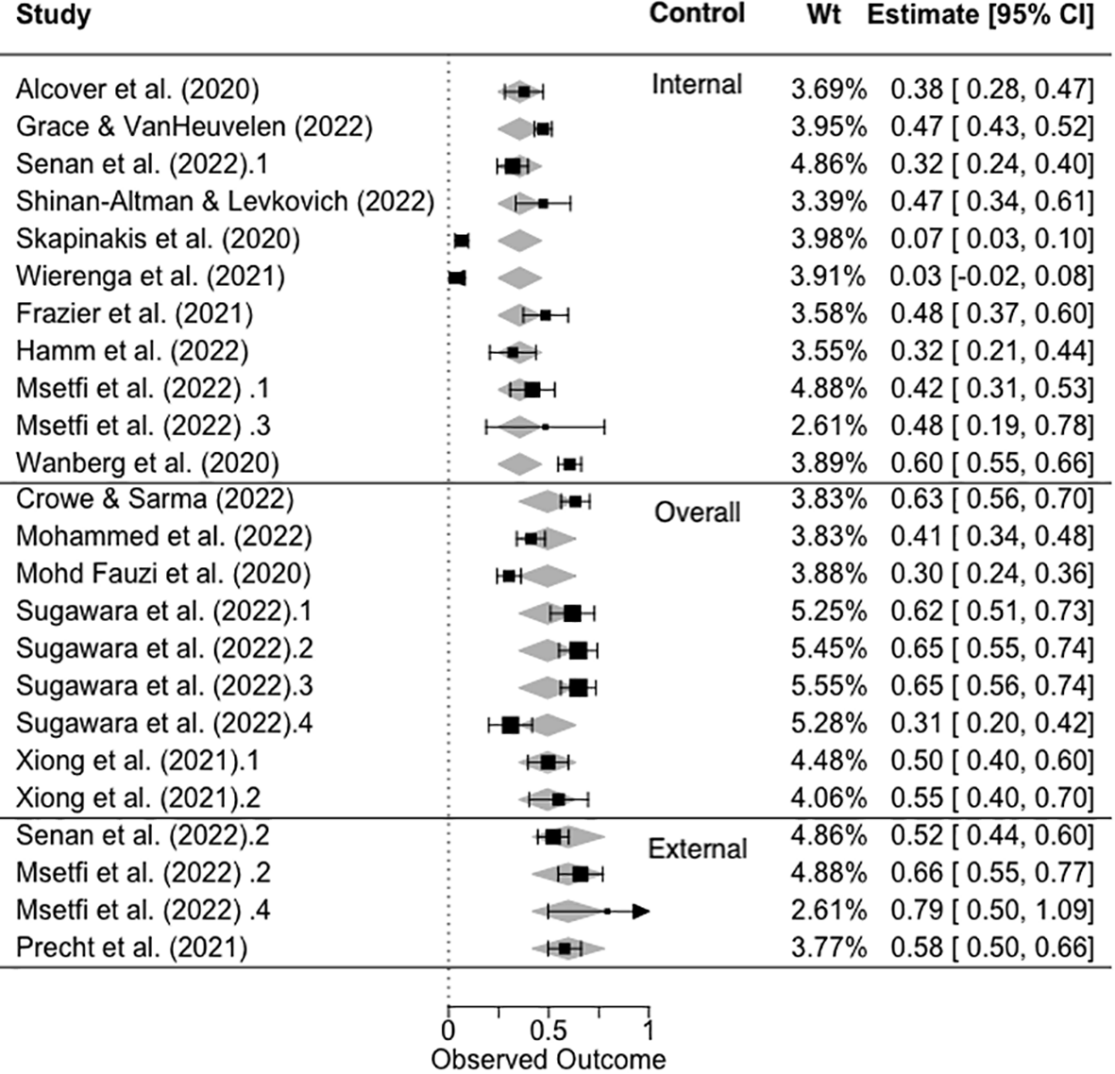
Forest plot showing the observed and fitted values of ES_Z_ as a function of control type (internal, overall, and external). NB. Black symbols = observed values, gray diamonds = fitted values.

We fitted a three-level meta-analysis model that included moderator control type (3: internal, overall, and external). The results showed that I^2^_Level 3_ = 46.86% of the total variation can be attributed to between-cluster, and I^2^_Level 2_ = 47.57% to within-cluster heterogeneity, and that there was significant residual heterogeneity in the model (*Q_E_* (21) = 569.93, *p* <.0001). The omnibus test of the control type was significant, *F*(2, 21) = 4.1465, *p* = 0.0304. The strongest association between control and depression was based on measuring ‘external’ control, estimate = .60 (*se* = .0867), *t*(21) = 6.89, *p* <.0001, with the association between ‘overall control’ and depression being weaker but not significantly so, estimate = .49 (*se* = .1103), *t*(21) = .94, *p* = .3591, and internal control having the weakest association with depression, estimate = .35 (*se* = .0905), and this was a significant difference, *t*(21)=2.68, *p* = .0141. The forest plot in **Figure 3** shows the *ES_Z_* ordered per control-type variable.

#### Q3. Does the effect size change as a function of pandemic indicators?

**Figure 4:**
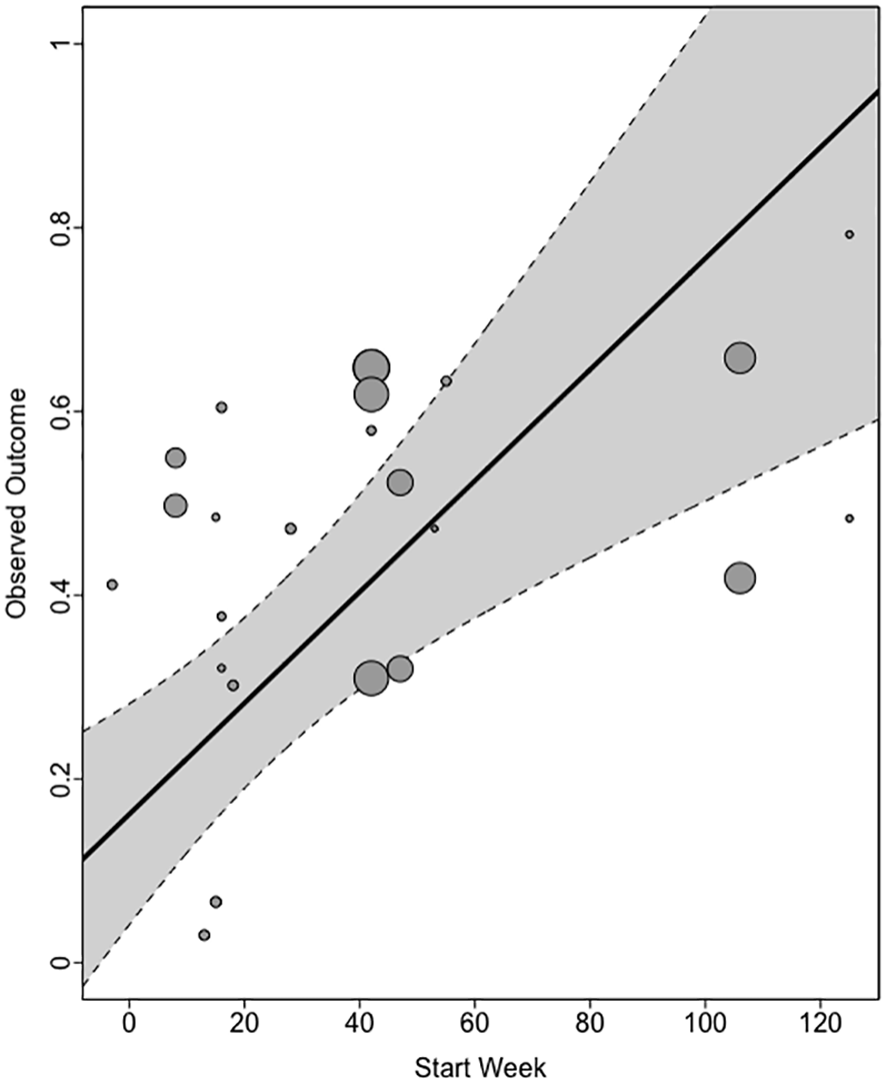
Relationship between start week and ES_Z_. Gray shading indicates the confidence limits of the model.

We fitted a three-level meta-analysis model, which included continuous moderators, start week, study duration, start week incidence (end week incidence was not included in the model due to the very high correlation with start week incidence *r* = .98) and the categorical moderator, continent (of data collection). The results showed that I^2^_Level 3_ = 48.63% of the total variation can be attributed to between-cluster heterogeneity, and I^2^_Level 2_ = 48.58% to within-cluster heterogeneity. There was significant residual heterogeneity in the model, *Q_E_* (20) = 607.94, *p* <.0001 The omnibus test of the moderators was significant, *F*(4, 20) = 13.93, *p <*0.0001. Only one pandemic indicator was a significant predictor of effect size; the start week was positively related to effect size, estimate = .006, se = .0017, *t* (21) = 3.65, *p* = .0016. Study duration (*p* = .60), start week incidence (*p* = .54), and continent, (*p* = .12), were not predictive of the effect size. This shows that, as the pandemic progressed over the roughly two-year time frame of these data collections, the association between sense of control and depression was stronger (see **Figure 4**).

## 4 Discussion

The results of this systematic review and meta-analysis show that most studies (80%) that were conducted very rapidly during the pandemic, measuring sense of control and depression, were of fair or good quality. The key biasing factors identified were primarily related to the aims of this review and the measurement of our variables of interest, as opposed to the aims of the original studies. This review showed that the relationship between sense of control and depression during the pandemic was medium to large. The meta-analysis further indicated that this was strongest for external and overall controls and weakest for internal controls. Finally, the only pandemic indicator that was predictive of the control–depression relationship was the study start week, showing that as the pandemic progressed from 2020 to mid-2022, the relationship between control and depression became stronger. We discuss these findings in relation to the previous evidence and the limitations of this study.

We predicted that the relationship between a sense of control and depression would be strong during the time frame of the pandemic. Consistent with this, the pooled estimate of the correlation was *r* = .41. We also observed that external control and overall control explained more variance in depression (external control *r* = .48–.68; overall control *r* = .29–.57) than internal control (internal control, *r* = .03–.54). Again, this finding is consistent with our predictions, although it does not tell us whether the observed pattern is significantly different from that of pre-pandemic studies. For example, Lachman and Weaver (3) reported that the results of several large-sample studies showed that the relationship between control and depression varied similarly (*r* external|constraints = |.24| to |.48|; *r* internal |mastery = |.19| to |.27|).

On the basis of similar findings, we agree with Infurna and Mayer (12), who argued that internal and external controls, although related, are distinct constructs and should be analyzed separately. This review, and many other examinations of the measurement of the sense of control, emphasize that there is considerable variation in the manner in which this important construct is conceptualized and measured across studies (11), for example (49), and whether control is decomposed into its constituent parts as evidence suggests it should be (12). Consistent with this view, the quality evaluation showed that the measurement of key variables, such as control, introduced significant bias into the review, and we therefore excluded four papers from quantitative analysis for this reason. Moreover, it was not possible to categorize all effect sizes included in the review as reflecting internal or external control measurements, rather some we categorized as ‘overall control.’ In our view, the use of amalgamated overall control measures is limited because it is clear that external control, related to an individual’s perception of the external barriers and restrictions they face, is more strongly related to mental and physical health (3).

A key contribution of the current work is to show, for the first time, that the relationship between control and depression changed over the course of the pandemic from weeks −3 to 130. The relationship grew stronger, indicating that control explained more of the variance in depression over time. Note that this evidence does not speak to absolute levels of depression or control, and how they vary during the pandemic. This is important, as evidence is equivocal on whether increases in depression, observed in the first wave of the pandemic, alleviated (20), continued to accumulate (50), and which groups were most vulnerable (51). Irrespective of this, there are several possible explanations for the growing relationship reported here. First, in the context of high depression levels among those who may not have typically experienced depression symptoms (5), the strength of the sense of control as an explanatory factor grew over the course of the pandemic. This may indicate that the loss of control during the pandemic resulted in this vulnerability. Second, the dynamic nature of the pandemic, involving repeated waves of the virus, lockdowns being imposed, lifted and reimposed, and requirements to be vaccinated, may have cumulatively eroded many people’s sense of control over their lives, particularly in relation to the salience of external obstacles. These findings are consistent with this explanation.

An alternative explanation for the ‘pandemic effect’ that must be considered is that effect sizes vary over time in a manner unrelated to the pandemic. In other words, it might be a mere coincidence that the time variable (study start week) occurred during the pandemic, and the effect sizes would have changed irrespective of this. Evidence for the alternative explanation is that no other pandemic indicators (covid incidence, continent) tested in this review predicted effect size, and that sense of control changes over time based on mini trends/or changes over the lifespan (6). Another challenge relates to the directionality of the relationship between control and depression. It is unclear from the data reported here whether sense of control is part of the causal pathway to depression or vice versa. Stimpson (52) reported that the relationship between changes in depression and control, from before to and 60-days after a flood disaster, had a reciprocal relationship, such that changes in the sense of control acted like a feedback loop influencing the depression caused by the flood. According to Stimpson, it was the flood experience that caused depression, rather than changes in the sense of control. Irrespective, all this evidence is consistent with the widely held view that the sense of control is important in determining responses to an environment that is constantly changing, whether it is due to floods, a pandemic, or other factors.

Our detailed quality assessment of the studies included in this review emphasized the importance of the measures used in studies as a distinguishing feature between those categorized as poor and fair to good. This meant that four studies and 14 effect sizes were excluded, reducing the noise in the data and reducing the power of our analyses. In addition, many of the effect sizes (11 of 24, 46%) were based on data collected before pandemic week 26 (6 months), so the latter part of the pandemic or after the pandemic is under-researched, and the post-pandemic effects are still unknown. A related point refers to another bias identified in the quality assessment: over-reliance on cross-sectional studies. As Mirowsky (6) described, inferring longitudinal trends from cross-sectional data is fraught with potential misinterpretation. Regarding both points—data mainly derived from early in the pandemic and the reliance on cross-sectional data—long-duration longitudinal studies can reveal distinct and informative trends. For example, one study based in the UK reported that depression decreased from the date of the first lockdown to 20 weeks afterwards (20). Longitudinal data from a nationally representative sample in Denmark showed initial improvements in mental health during the first lockdown; however, mental health deteriorated as the pandemic progressed (50). This contrast demonstrates the need for caution when interpreting time-based trends, as in the current review.

## 5 Conclusion

The general consensus is that a pandemic has a negative impact on mental health (53, 54). This review confirms that changes in sense of control, particularly the perception of external constraints, played an increasingly large role in depression severity as the pandemic progressed. Future research should measure the components of sense of control to better inform interventions. At the policy level, the clinical implication is that public health restrictions should be designed to provide as much autonomy as possible, preserve people’s feelings of control, and protect their mental health. Good quality longitudinal studies are also important, as the jury is still out regarding the long-term recovery from pandemic-induced mental health deterioration and the long-term effects of the pandemic.

## Author contributions

RM: Conceptualization, Data curation, Formal analysis, Methodology, Resources, Supervision, Visualization, Writing – original draft, Writing – review & editing. DK: Formal analysis, Methodology, Validation, Visualization, Writing – original draft, Writing – review & editing. YH: Data curation, Formal analysis, Project administration, Validation, Visualization, Writing – original draft, Writing – review & editing.

## References

[1] Mirowsky J Ross CE. Control or defense? Depression and the sense of control over good and bad outcomes. J Health Soc Behav (1990) 31:71–86. doi: 10.2307/2137046 2313078

[2] Steptoe A Tsuda A Tanaka Y Wardle J. Depressive symptoms, socio-economic background, sense of control, and cultural factors in university students from 23 countries. Int J Behav Med (2007) 14:97–107. doi: 10.1007/bf03004175 17926438

[3] Lachman ME Weaver SL. The sense of control as a moderator of social class differences in health and well-being. JPSP (1998) 74:763. doi: 10.1037/0022-3514.74.3.763 9523418

[4] Maier SF Seligman ME. Learned helplessness: Theory and evidence. J Exp Psychology-General (1976) 105:3–46. doi: 10.1037/0096-3445.105.1.3

[5] Bueno-Notivol J Gracia-Garcia P Olaya B Lasheras I Lopez-Anton R Santabarbara J. Prevalence of depression during the COVID-19 outbreak: A meta-analysis of community-based studies. Int J Clin Health Psychol (2021) 21. doi: 10.1016/j.ijchp.2020.07.007 PMC7458054 32904715

[6] Mirowsky J. Depression and the sense of control: Aging vectors, trajectories, and trends. J Health Soc Behav (2013) 54:407–25. doi: 10.1177/0022146513499022 PMC3856322 24311752

[7] Mystakidou K Tsilika E Parpa E Galanos A. The influence of sense of control and cognitive functioning in older cancer patients’ depression. Psycho-Oncology (2015) 24:311–7. doi: 10.1002/pon.3642 25082558

[8] Wallhagen MI Brod M. Perceived control and well-being in Parkinson’s disease. Western J Nurs Res (1997) 19:11–25. doi: 10.1177/019394599701900102 9030036

[9] Craig A Hancock K Chang E Dickson H. The effectiveness of group psychological intervention in enhancing perceptions of control following spinal cord injury. Aust N Z. J Psychiatry (1998) 32:112–8. doi: 10.1046/j.1440-1614.1998.00376.x 9565192

[10] Thompson SC Sobolew-Shubin A Galbraith ME Schwankovsky L Cruzen D. Maintaining perceptions of control: finding perceived control in low-control circumstances. JPSP (1993) 64:293. doi: 10.1037/0022-3514.64.2.293 8433275

[11] Skinner EA. A guide to constructs of control. JPSP (1996) 71:549–70. doi: 10.1037/0022-3514.71.3.549 8831161

[12] Infurna FJ Mayer A. The effects of constraints and mastery on mental and physical health: Conceptual and methodological considerations. Psychol Aging (2015) 30:432. doi: 10.1037/a0039050 25938243 PMC4451433

[13] Infurna FJ Kappes C Fraire N. Long-term antecedents of constraints and mastery: Findings from the Health and Retirement Study. Psychol Aging (2018) 33:965–74. doi: 10.1037/pag0000281 30198734

[14] Schafer KM Lieberman A Sever AC Joiner T. Prevalence rates of anxiety, depressive, and eating pathology symptoms between the pre- and peri-COVID-19 eras: A meta-analysis. J Affect Disord (2022) 298:364–72. doi: 10.1016/j.jad.2021.10.115 PMC8593520 34740748

[15] Hajek A. Prevalence and determinants of probable depression and anxiety during the COVID-19 pandemic in seven countries: Longitudinal evidence from the European COvid Survey (ECOS). J Affect Disord (2022) 299:517–24. doi: 10.1016/j.jad.2021.12.029 PMC8684990 34920039

[16] Brailovskaia J Margraf J. Predicting adaptive and maladaptive responses to the Coronavirus (COVID-19) outbreak: A prospective longitudinal study. Int J Clin Health Psychol (2020) 20:183–91. doi: 10.1016/j.ijchp.2020.06.002 PMC7321043 32837518

[17] Xiong P Ming W-k Zhang C Bai J Luo C Cao W. Factors influencing mental health among Chinese medical and non-medical students in the early stage of the COVID-19 pandemic. Front Public Health (2021) 9:603331. doi: 10.3389/fpubh.2021.603331 34095044 PMC8172592

[18] Gan Y. Immediate and delayed psychological effects of province-wide lockdown and personal quarantine during the COVID-19 outbreak in China. Psychol Med (2022) 52:1321–32. doi: 10.1017/S0033291720003116 PMC7450230 32787981

[19] Senan S Halbrook Y Kornbrot DE Msetfi RM. Depression symptoms and the perception of public health restrictions during the COVID-19 pandemic in Saudi Arabia: The protective effect of sense of control. Prev Med Rep (2022), 101836. doi: 10.1016/j.pmedr.2022.101836 35601456 PMC9113956

[20] Fancourt D Steptoe A Bu FF. Trajectories of anxiety and depressive symptoms during enforced isolation due to COVID-19 in England: a longitudinal observational study. Lancet Psychiat (2021) 8:141–9. doi: 10.1016/S2215-0366(20)30482-X PMC7820109 33308420

[21] Page MJ. The PRISMA 2020 statement: an updated guideline for reporting systematic reviews. Syst Rev-London (2021) 10. doi: 10.1186/s13643-021-01626-4 PMC8008539 33781348

[22] Haddaway NR Page MJ Pritchard CC McGuinness LA. PRISMA2020: An R package and Shiny app for producing PRISMA 2020-compliant flow diagrams, with interactivity for optimised digital transparency and Open Synthesis. Campbell Syst Rev 18:(2022). doi: 10.1002/cl2.1230 PMC8958186 36911350

[23] Covidence systematic review software. Melbourne, Australia (2022). Available at: www.covidence.org. V. H. I.

[24] Rotter JB. Generalized expectancies for internal versus external control of reinforcement. psychol Monographs: Gen Appl (1966) 80:1. doi: 10.1037/h0092976 5340840

[25] Lazarus RS Folkman S. Stress, appraisal, and coping. Springer publishing company (1984).

[26] Ryon HS Gleason ME. The role of locus of control in daily life. Pers Soc Psychol Bull (2014) 40:121–31. doi: 10.1177/0146167213507087 24107710

[27] NIH National Heart Lung, Blood Institute. NIH quality assessment tool for observational cohort and cross-sectional studies. Study quality assessment tools (2013). Available online at: https://www.nhlbi.nih.gov/health-topics/study-quality-assessment-tools (accessed January 2023).

[28] Alcover CM. Group membership and social and personal identities as psychosocial coping resources to psychological consequences of the COVID-19 confinement. Int J Environ Res Public Health (2020) 17. doi: 10.3390/ijerph17207413 PMC7601487 33053738

[29] Crowe S Sarma K. Coping with Covid-19: Stress, control and coping among pregnant women in Ireland during the Covid-19 pandemic. BMC Pregnancy Childbirth (2022) 22:1–12. doi: 10.1186/s12884-022-04579-1 35365093 PMC8972984

[30] Curl AL Wolf KE. The impact of COVID-19 on depressive symptoms and loneliness for middle-aged and older adults. Sustainability (2022) 14:6316. doi: 10.3390/su14106316

[31] Frazier P Liu Y Asplund A Meredith L Nguyen-Feng VN. US college student mental health and COVID-19: Comparing pre-pandemic and pandemic timepoints. J Am Coll Health (2021) 1–11. doi: 10.1080/07448481.2021.1987247 34762560

[32] Grace MK VanHeuvelen JS. Psychosocial coping resources and the toll of COVID-19 bereavement. Soc Ment Health (2022) 1–23. doi: 10.1177/21568693221113221

[33] Hamm JM Tan JX Barlow MA Delaney RL Duggan KA. Goal adjustment capacities in uncontrollable life circumstances: Benefits for psychological well-being during COVID-19. Motivation Emotion (2022), 1–17. doi: 10.1007/s11031-022-09941-6 PMC9124288 35633867

[34] Kondo A Abuliezi R Niitsu K Naruse K Oki T Ota E. Factors related to mental health effect among nursing students in Japan and the United States during the coronavirus pandemic: A cross-sectional study. Int J Ment Health Nurs (2022). doi: 10.1111/inm.13075 PMC9538421 36184845

[35] Mohammed HM Soliman SM Abdelrahman AA Ibrahim AK. Depressive symptoms and its correlates among medical students in Upper Egypt. Middle East Curr Psychiatry (2022) 29:1–9. doi: 10.1186/s43045-022-00231-y

[36] Mohd Fauzi MF. Doctors’ mental health in the midst of COVID-19 pandemic: The roles of work demands and recovery experiences. Int J Environ Res Public Health (2020) 17:7340. doi: 10.3390/ijerph17197340 33050004 PMC7579590

[37] Msetfi R Kornbrot D Halbrook YJ Senan S. Sense of control and depression during public health restrictions and the COVID-19 pandemic. Int J Environ Res Public Health (2022) 19:14429. doi: 10.3390/ijerph192114429 36361309 PMC9658609

[38] Precht L-M Margraf J Stirnberg J Brailovskaia J. It’s all about control: Sense of control mediates the relationship between physical activity and mental health during the COVID-19 pandemic in Germany. Curr Psychol (2021), 1–9. doi: 10.1007/s12144-021-02303-4 PMC8527308 34690477

[39] Sahni PS Singh K Sharma N Garg R. Yoga an effective strategy for self-management of stress-related problems and wellbeing during COVID19 lockdown: A cross-sectional study. PloS One (2021) 16:e0245214. doi: 10.1371/journal.pone.0245214 33566848 PMC7875402

[40] Shinan-Altman S Levkovich I. Are personal resources and perceived stress associated with psychological outcomes among Israeli teachers during the third COVID-19 lockdown? Int J Environ Res Public Health (2022) 19:5634. doi: 10.3390/ijerph19095634 35565027 PMC9099812

[41] Skapinakis P. Depression and its relationship with coping strategies and illness perceptions during the COVID-19 lockdown in Greece: a cross-sectional survey of the population. Depression Res Treat (2020) 2020. doi: 10.1155/2020/3158954 PMC7450302 32908697

[42] Sugawara D. Mental health and psychological resilience during the COVID-19 pandemic: A cross-cultural comparison of Japan, Malaysia, China, and the US. J Affect Disord (2022) 311:500–7. doi: 10.1016/j.jad.2022.05.032 PMC9090817 35561884

[43] Van Mulukom V Muzzulini B Rutjens BT Van Lissa CJ Farias M. The psychological impact of threat and lockdowns during the COVID-19 pandemic: Exacerbating factors and mitigating actions. Trans Behav Med (2021) 11:1318–29. doi: 10.1093/tbm/ibab072 PMC8420639 34155522

[44] Wanberg CR Csillag B Douglass RP Zhou L Pollard MS. Socioeconomic status and well-being during COVID-19: A resource-based examination. J Appl Psychol (2020) 105:1382. doi: 10.1037/apl0000831 33090858 PMC7899012

[45] Wierenga KL Moore SE Pressler SJ Hacker ED Perkins SM. Associations between COVID-19 perceptions, anxiety, and depressive symptoms among adults living in the United States. Nurs Outlook (2021) 69:755–66. doi: 10.1016/j.outlook.2021.03.020 PMC8530452 33894985

[46] ECDC. European Centre for Disease Prevention and Control. (2005-2022). Available online at: https://www.ecdc.europa.eu/en/publications-data/data-daily-new-cases-covid-19-eueea-country.

[47] Viechtbauer W. Conducting meta-analyses in R with the metafor package. J Stat software (2010) 36:1–48. doi: 10.18637/jss.v036.i03

[48] Cohen J. Statistical power analysis for the behavioral sciences. New York: Academic press (2013).

[49] Mirowsky J Ross CE. Eliminating defense and agreement bias from measures of the sense of control: A 2 x 2 index. SPsy (1991), 127–45. doi: 10.2307/2786931

[50] Cardona M Andersen LH Fallesen P Bruckner TA. Stress/depression across the COVID-19 pandemic in Denmark. BMC Public Health (2023) 23:169. doi: 10.1186/s12889-023-15129-5 36698122 PMC9875528

[51] Pedersen MT. Time trends in mental health indicators during the initial 16 months of the COVID-19 pandemic in Denmark. BMC Psychiatry (2022) 22:1–13. doi: 10.1186/s12888-021-03655-8 35012486 PMC8743441

[52] Stimpson JP. Prospective evidence for a reciprocal relationship between sense of control and depressive symptoms following a flood. Stress Health: J Int Soc Invest Stress (2006) 22:161–6. doi: 10.1002/smi.1091

[53] Leung CMC Ho MK Bharwani AA Cogo-Moreira H Wang Y Chow MSC. Mental disorders following COVID-19 and other epidemics: a systematic review and meta-analysis. Trans Psychiatry (2022) 12:205. doi: 10.1038/s41398-022-01946-6 PMC9110635 35581186

[54] Wirkner J. Mental health in times of the COVID-19 pandemic. Eur Psychol (2022). doi: 10.1027/1016-9040/a000465

