# Supplementary material for: The association between the sense of control and depression during the COVID-19 pandemic: a systematic review and meta-analysis
Source: Front Psychiatry. 2024 Feb 13;15:1323306. doi: 10.3389/fpsyt.2024.1323306 (PMC10897004; doi:10.3389/fpsyt.2024.1323306)
Supplement: Supplementary file 1 [file DataSheet_1.zip › 1a_OverallAnalysisWithAndWithoutQuality.docx]

**Analysis for results2**

**Using data from 38 rows**

df=SPSS_38rows_070323

yi=(df$Z_r2)

vi=(df$Var_Z)

z=(df$Study_num)

a=(df$Quality)

a2=(df$QUALITY_cat)

b=(df$study1_20)

b2=(df$esid)

b3=(df$esid_unique)

c=(df$Country_StudyID)

d=(df$Control_var_num)

e=(df$StartWeek)

f=(df$EndWeek)

g=(df$dif_week)

h=(df$Author)

i=(df$Continent)

j=(df$Startweekincidence)

k=(df$Endweekincidence)

full.model <- rma.mv(yi, vi, slab=df$Author, random=~1 | b/b3,

data=df, method="REML",

test="z", dfs="residual", level=95, cvvc=TRUE, sparse=FALSE, verbose=FALSE, digits=4)

summary(full.model)

Multivariate Meta-Analysis Model (k = 38; method: REML)

logLik Deviance AIC BIC AICc

16.0644 -32.1287 -26.1287 -21.2960 -25.4014

Variance Components:

estim sqrt nlvls fixed factor

sigma^2.1 0.0285 0.1688 20 no b

sigma^2.2 0.0075 0.0868 38 no b/b3

Test for Heterogeneity:

Q(df = 37) = 1119.8299, p-val < .0001

Model Results:

estimate se zval pval ci.lb ci.ub

0.3852 0.0422 9.1210 <.0001 0.3025 0.4680 ***

---

Signif. codes: 0 ‘***’ 0.001 ‘**’ 0.01 ‘*’ 0.05 ‘.’ 0.1 ‘ ’ 1

df=FairToGood2_070323

yi=(df$Z_r2)

vi=(df$Var_Z)

z=(df$Study_num)

a=(df$Quality)

a2=(df$Quality_cat)

b=(df$study1_20)

b2=(df$esid)

b3=(df$esid_unique)

c=(df$Country_StudyID)

d=(df$Control_var_num)

e=(df$StartWeek)

f=(df$EndWeek)

g=(df$dif_week)

h=(df$Author)

i=(df$Continent)

j=(df$Startweekincidence)

k=(df$Endweekincidence)

full.model24 <- rma.mv(yi, vi, slab=df$Author, random=~1 | b/b3,

data=df, method="REML",

test="z", dfs="residual", level=95, cvvc=TRUE, sparse=FALSE, verbose=FALSE, digits=4)

summary(full.model24)

Multivariate Meta-Analysis Model (k = 24; method: REML)

logLik Deviance AIC BIC AICc

6.7060 -13.4120 -7.4120 -4.0055 -6.1489

Variance Components:

estim sqrt nlvls fixed factor

sigma^2.1 0.0113 0.1062 16 no b

sigma^2.2 0.0204 0.1428 24 no b/b3

Test for Heterogeneity:

Q(df = 23) = 798.9004, p-val < .0001

Model Results:

estimate se zval pval ci.lb ci.ub

0.4402 0.0423 10.3955 <.0001 0.3572 0.5231 ***

---

Signif. codes: 0 ‘***’ 0.001 ‘**’ 0.01 ‘*’ 0.05 ‘.’ 0.1 ‘ ’ 1

**Add Quality into the analysis (38 rows)**

df=SPSS_38rows_070323

yi=(df$Z_r2)

vi=(df$Var_Z)

z=(df$Study_num)

a=(df$Quality)

a2=(df$QUALITY_cat)

b=(df$study1_20)

b2=(df$esid)

b3=(df$esid_unique)

c=(df$Country_StudyID)

d=(df$Control_var_num)

e=(df$StartWeek)

f=(df$EndWeek)

g=(df$dif_week)

h=(df$Author)

i=(df$Continent)

j=(df$Startweekincidence)

k=(df$Endweekincidence)

full.modelqual <- rma.mv(yi, vi, slab=df$Author, random=~1 | b/b3, mods=~a2,

data=df, method="REML",

test="z", dfs="residual", level=95, cvvc=TRUE, sparse=FALSE, verbose=FALSE, digits=4, sigma2 = c(0, NA))

summary(full.modelqual)

**Multivariate Meta-Analysis Model (k = 38; method: REML)**

logLik Deviance AIC BIC AICc

11.7295 -23.4591 -15.4591 -9.2377 -14.1257

Variance Components:

estim sqrt nlvls fixed factor

sigma^2.1 0.0000 0.0000 20 yes b

sigma^2.2 0.0276 0.1663 38 no b/b3

Test for Residual Heterogeneity:

QE(df = 35) = 1015.3798, p-val < .0001

Test of Moderators (coefficients 2:3):

QM(df = 2) = 5.6578, p-val = 0.0591

Model Results:

estimate se zval pval ci.lb ci.ub

intrcpt 0.4349 0.0418 10.4054 <.0001 0.3530 0.5169 ***

a2Good 0.0912 0.0815 1.1189 0.2632 -0.0686 0.2510

a2Poor -0.0988 0.0623 -1.5858 0.1128 -0.2208 0.0233

---

Signif. codes: 0 ‘***’ 0.001 ‘**’ 0.01 ‘*’ 0.05 ‘.’ 0.1 ‘ ’ 1

**Mods with Qual (for 24 rows)**

df=FairToGood2_070323

yi=(df$Z_r2)

vi=(df$Var_Z)

z=(df$Study_num)

a=(df$Quality)

b=(df$study1_20)

b2=(df$esid)

b3=(df$esid_unique)

c=(df$Country_StudyID)

d=(df$Control_var_num)

e=(df$StartWeek)

f=(df$EndWeek)

g=(df$dif_week)

h=(df$Author)

i=(df$Continent)

j=(df$Startweekincidence)

k=(df$Endweekincidence)

full.model24qual <- rma.mv(yi, vi, slab=df$Author, random=~1 | b/b3, mods=~a2, intercept=TRUE,

data=df, method="REML",

test="z", dfs="residual", level=95, cvvc=TRUE, sparse=FALSE, verbose=FALSE, digits=4, sigma2 = c(0, NA))

summary(full.model24qual)

Multivariate Meta-Analysis Model (k = 24; method: REML)

logLik Deviance AIC BIC AICc

6.2705 -12.5410 -6.5410 -3.2679 -5.2076

Variance Components:

estim sqrt nlvls fixed factor

sigma^2.1 0.0000 0.0000 16 yes b

sigma^2.2 0.0305 0.1748 24 no b/b3

Test for Residual Heterogeneity:

QE(df = 22) = 708.8150, p-val < .0001

Test of Moderators (coefficient 2):

QM(df = 1) = 1.1559, p-val = 0.2823

Model Results:

estimate se zval pval ci.lb ci.ub

intrcpt 0.4353 0.0438 9.9383 <.0001 0.3494 0.5211 ***

a2Good 0.0915 0.0851 1.0751 0.2823 -0.0753 0.2584

---

Signif. codes: 0 ‘***’ 0.001 ‘**’ 0.01 ‘*’ 0.05 ‘.’ 0.1 ‘ ’ 1

$results

% of total variance I2

Level 1 4.801751 ---

Level 2 95.198249 95.2

Level 3 0.000000 0

On the basis of all of this we have decided to remove the Poor studies from the analysis.
