# Supplementary material for: The association between the sense of control and depression during the COVID-19 pandemic: a systematic review and meta-analysis
Source: Front Psychiatry. 2024 Feb 13;15:1323306. doi: 10.3389/fpsyt.2024.1323306 (PMC10897004; doi:10.3389/fpsyt.2024.1323306)
Supplement: Supplementary file 1 [file DataSheet_1.zip › 1b_Model24WithAndWIthoutNest.docx]

**Analysis for results2**

**Using data from 38 rows**

df=SPSS_38rows_070323

yi=(df$Z_r2)

vi=(df$Var_Z)

z=(df$Study_num)

a=(df$Quality)

a2=(df$QUALITY_cat)

b=(df$study1_20)

b2=(df$esid)

b3=(df$esid_unique)

c=(df$Country_StudyID)

d=(df$Control_var_num)

e=(df$StartWeek)

f=(df$EndWeek)

g=(df$dif_week)

h=(df$Author)

i=(df$Continent)

j=(df$Startweekincidence)

k=(df$Endweekincidence)

full.model <- rma.mv(yi, vi, slab=df$Author, random=~1 | b/b3,

data=df, method="REML",

test="z", dfs="residual", level=95, cvvc=TRUE, sparse=FALSE, verbose=FALSE, digits=4)

summary(full.model)

Multivariate Meta-Analysis Model (k = 38; method: REML)

logLik Deviance AIC BIC AICc

16.0644 -32.1287 -26.1287 -21.2960 -25.4014

Variance Components:

estim sqrt nlvls fixed factor

sigma^2.1 0.0285 0.1688 20 no b

sigma^2.2 0.0075 0.0868 38 no b/b3

Test for Heterogeneity:

Q(df = 37) = 1119.8299, p-val < .0001

Model Results:

estimate se zval pval ci.lb ci.ub

0.3852 0.0422 9.1210 <.0001 0.3025 0.4680 ***

---

Signif. codes: 0 ‘***’ 0.001 ‘**’ 0.01 ‘*’ 0.05 ‘.’ 0.1 ‘ ’ 1

Multivariate Meta-Analysis Model (k = 24; method: REML)

logLik Deviance AIC BIC AICc

6.7060 -13.4120 -7.4120 -4.0055 -6.1489

Variance Components:

estim sqrt nlvls fixed factor

sigma^2.1 0.0113 0.1062 16 no b

sigma^2.2 0.0204 0.1428 24 no b/b3

Test for Heterogeneity:

Q(df = 23) = 798.9004, p-val < .0001

Model Results:

estimate se zval pval ci.lb ci.ub

0.4402 0.0423 10.3955 <.0001 0.3572 0.5231 ***

---

Signif. codes: 0 ‘***’ 0.001 ‘**’ 0.01 ‘*’ 0.05 ‘.’ 0.1 ‘ ’ 1

**SAME MODEL NOT NESTED**

Multivariate Meta-Analysis Model (k = 24; method: REML)

logLik Deviance AIC BIC AICc

-9.7597 19.5194 23.5194 25.7904 24.1194

Variance Components:

estim sqrt nlvls fixed factor

sigma^2 0.0309 0.1758 16 no b

Test for Heterogeneity:

Q(df = 23) = 798.9004, p-val < .0001

Model Results:

estimate se zval pval ci.lb ci.ub

0.4244 0.0451 9.4178 <.0001 0.3361 0.5127 ***

---

Signif. codes: 0 ‘***’ 0.001 ‘**’ 0.01 ‘*’ 0.05 ‘.’ 0.1 ‘ ’ 1

**> anova(full.model24, full.modelnot)**

df AIC BIC AICc logLik LRT pval QE

Full 3 -7.4120 -4.0055 -6.1489 6.7060 798.9004

Reduced 2 23.5194 25.7904 24.1194 -9.7597 32.9314 <.0001 798.9004

**Add Quality into the analysis (38 rows)**

df=SPSS_38rows_070323

yi=(df$Z_r2)

vi=(df$Var_Z)

z=(df$Study_num)

a=(df$Quality)

a2=(df$QUALITY_cat)

b=(df$study1_20)

b2=(df$esid)

b3=(df$esid_unique)

c=(df$Country_StudyID)

d=(df$Control_var_num)

e=(df$StartWeek)

f=(df$EndWeek)

g=(df$dif_week)

h=(df$Author)

i=(df$Continent)

j=(df$Startweekincidence)

k=(df$Endweekincidence)

full.modelqual <- rma.mv(yi, vi, slab=df$Author, random=~1 | b/b3, mods=(a1),

data=df, method="REML",

test="z", dfs="residual", level=95, cvvc=TRUE, sparse=FALSE, verbose=FALSE, digits=4, sigma2 = c(0, NA))

summary(full.modelqual)

Multivariate Meta-Analysis Model (k = 38; method: REML)

logLik Deviance AIC BIC AICc

12.8817 -25.7635 -19.7635 -15.0129 -19.0135

Variance Components:

estim sqrt nlvls fixed factor

sigma^2.1 0.0000 0.0000 20 yes b

sigma^2.2 0.0263 0.1622 38 no b/b3

Test for Residual Heterogeneity:

QE(df = 36) = 1049.8041, p-val < .0001

Test of Moderators (coefficient 2):

QM(df = 1) = 6.5681, p-val = 0.0104

Model Results:

estimate se zval pval ci.lb ci.ub

intrcpt 0.2361 0.0742 3.1816 0.0015 0.0906 0.3815 **

mods 0.0973 0.0380 2.5628 0.0104 0.0229 0.1717 *

---

Signif. codes: 0 ‘***’ 0.001 ‘**’ 0.01 ‘*’ 0.05 ‘.’ 0.1 ‘ ’ 1

**Mods with Qual (for 24 rows)**

df=FairToGood2_070323

yi=(df$Z_r2)

vi=(df$Var_Z)

z=(df$Study_num)

a=(df$Quality)

b=(df$study1_20)

b2=(df$esid)

b3=(df$esid_unique)

c=(df$Country_StudyID)

d=(df$Control_var_num)

e=(df$StartWeek)

f=(df$EndWeek)

g=(df$dif_week)

h=(df$Author)

i=(df$Continent)

j=(df$Startweekincidence)

k=(df$Endweekincidence)

full.model24qual <- rma.mv(yi, vi, slab=df$Author, random=~1 | b/b3, mods=(a), intercept=TRUE,

data=df, method="REML",

test="z", dfs="residual", level=95, cvvc=TRUE, sparse=FALSE, verbose=FALSE, digits=4, sigma2 = c(0, NA))

summary(full.model24qual)

Multivariate Meta-Analysis Model (k = 24; method: REML)

logLik Deviance AIC BIC AICc

6.5695 -13.1390 -7.1390 -3.8659 -5.8057

Variance Components:

estim sqrt nlvls fixed factor

sigma^2.1 0.0000 0.0000 16 yes b

sigma^2.2 0.0296 0.1721 24 no b/b3

Test for Residual Heterogeneity:

QE(df = 22) = 673.5703, p-val < .0001

Test of Moderators (coefficient 2):

QM(df = 1) = 1.8105, p-val = 0.1784

Model Results:

estimate se zval pval ci.lb ci.ub

intrcpt 0.2102 0.1888 1.1128 0.2658 -0.1600 0.5803

mods 0.1080 0.0802 1.3455 0.1784 -0.0493 0.2652

---

Signif. codes: 0 ‘***’ 0.001 ‘**’ 0.01 ‘*’ 0.05 ‘.’ 0.1 ‘ ’ 1

On the basis of all of this we have decided to remove the Poor studies from the analysis.
