# Supplementary material for: The association between the sense of control and depression during the COVID-19 pandemic: a systematic review and meta-analysis
Source: Front Psychiatry. 2024 Feb 13;15:1323306. doi: 10.3389/fpsyt.2024.1323306 (PMC10897004; doi:10.3389/fpsyt.2024.1323306)

Analysis of results 3 (control)

Theoretical Analysis

df=FairToGood2_070323

yi=(df$Z_r2)

vi=(df$Var_Z)

z=(df$Study_num)

a=(df$Quality)

b=(df$study1_20)

b2=(df$esid)

b3=(df$esid_unique)

c=(df$Country_StudyID)

d=(df$Control_var_num)

d2=(df$control_type)

e=(df$StartWeek)

f=(df$EndWeek)

g=(df$dif_week)

h=(df$Author)

i=(df$Continent)

j=(df$Startweekincidence)

k=(df$Endweekincidence)

full.modelcontrol <- rma.mv(yi, vi, slab=df$Author, random=~1 | b/b3, intercept=TRUE,

data=df, method="REML", mods = ~ control_type,

test="t", dfs="residual", level=95, cvvc=TRUE, sparse=FALSE, verbose=FALSE, digits=4)

summary(full.modelcontrol)

Multivariate Meta-Analysis Model (k = 24; method: REML)

logLik Deviance AIC BIC AICc

8.8476 -17.6951 -7.6951 -2.4725 -3.6951

Variance Components:

estim sqrt nlvls fixed factor

sigma^2.1 0.0130 0.1138 16 no b

sigma^2.2 0.0132 0.1147 24 no b/b3

Test for Residual Heterogeneity:

QE(df = 21) = 569.9291, p-val < .0001

c

Test of Moderators (coefficients 2:3):

F(df1 = 2, df2 = 21) = 4.1465, p-val = 0.0304

Model Results:

estimate se tval df pval ci.lb ci.ub

intrcpt 0.5974 0.0867 6.8882 21 <.0001 0.4170 0.7777 ***

control_typeinternal -0.2422 0.0905 -2.6765 21 0.0141 -0.4305 -0.0540 *

control_typeOverall -0.1034 0.1103 -0.9376 21 0.3591 -0.3327 0.1259

---

Signif. codes: 0 ‘***’ 0.001 ‘**’ 0.01 ‘*’ 0.05 ‘.’ 0.1 ‘ ’ 1

---

i2 <- var.comp(full.modelpandemic)

print (i2)

$results

% of total variance I2

Level 1 5.572193 ---

Level 2 47.568012 47.57

Level 3 46.859795 46.86

$totalI2

[1] 94.42781


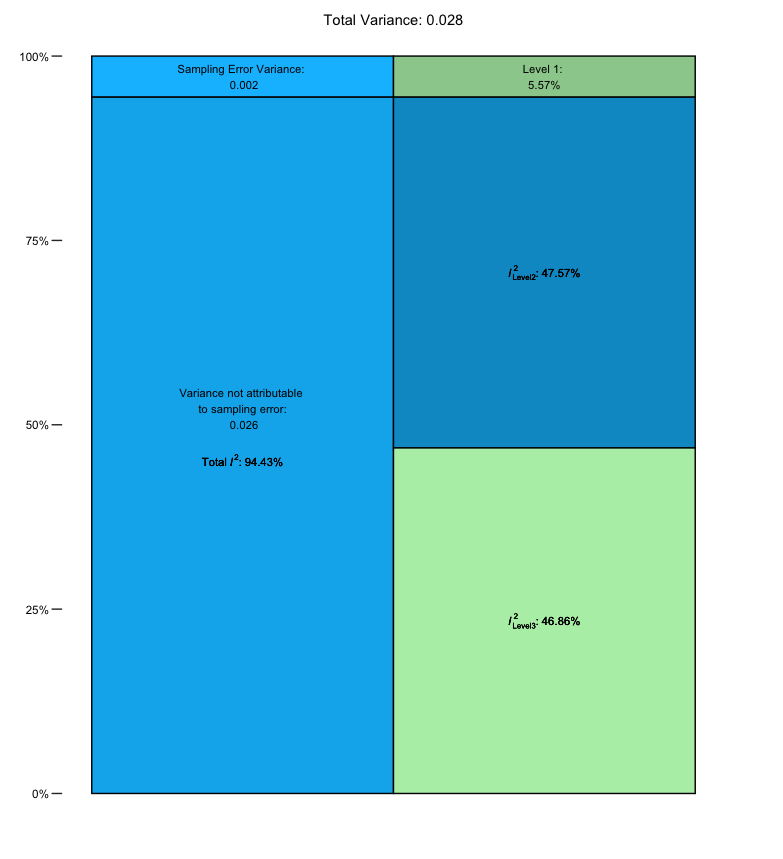

Supplement: Supplementary file 1 [file DataSheet_1.zip › 2_AnalysisControlModeRcode.docx]
