# Supplementary material for: The association between the sense of control and depression during the COVID-19 pandemic: a systematic review and meta-analysis
Source: Front Psychiatry. 2024 Feb 13;15:1323306. doi: 10.3389/fpsyt.2024.1323306 (PMC10897004; doi:10.3389/fpsyt.2024.1323306)
Supplement: Supplementary file 1 [file DataSheet_1.zip › 3_PandemicAnalysisRcode.docx]

Highlighted bit

R-code- Make sure that you have the following packages active – Meta, Metafor, Metadata, methods, ggplot2, graphics, numderiv, haven, graphics, matrix, clubsandwich;

df=FairToGood2_070323

yi=(df$Z_r2)

vi=(df$Var_Z)

z=(df$Study_num)

a=(df$Quality)

b=(df$study1_20)

b2=(df$esid)

b3=(df$esid_unique)

c=(df$Country_StudyID)

d=(df$Control_var_num)

d2=(df$control_type)

e=(df$StartWeek)

f=(df$EndWeek)

g=(df$dif_week)

h=(df$Author)

i=(df$Continent)

j=(df$Startweekincidence)

k=(df$Endweekincidence)

l=(df$Int_startWek_Incidence)

m=(df$Country)

full.modelpandemic <- rma.mv(yi, vi, slab=df$Author, random=~1 | b/b3, mods=~ e + g + j + i -1,

data=df, method="REML",

test="t", dfs="residual", level=95, cvvc=TRUE, sparse=FALSE, verbose=FALSE, digits=4)

summary(full.modelpandemic)

Multivariate Meta-Analysis Model (k = 24; method: REML)

logLik Deviance AIC BIC AICc

1.4366 -2.8732 9.1268 15.1012 15.5884

Variance Components:

estim sqrt nlvls fixed factor

sigma^2.1 0.0268 0.1639 16 no b

sigma^2.2 0.0268 0.1638 24 no b/b3

Test for Residual Heterogeneity:

QE(df = 20) = 607.9434, p-val < .0001

Test of Moderators (coefficients 1:4):

F(df1 = 4, df2 = 20) = 13.9274, p-val < .0001

Model Results:

estimate se tval df pval ci.lb ci.ub

e 0.0060 0.0017 3.6489 20 0.0016 0.0026 0.0095 **

g 0.0079 0.0145 0.5485 20 0.5894 -0.0222 0.0381

j -0.0000 0.0001 -0.6216 20 0.5412 -0.0002 0.0001

i 0.0667 0.0417 1.6015 20 0.1249 -0.0202 0.1536

---

Signif. codes: 0 ‘***’ 0.001 ‘**’ 0.01 ‘*’ 0.05 ‘.’ 0.1 ‘ ’ 1

$results

% of total variance I2

Level 1 2.790512 ---

Level 2 48.579268 48.58

Level 3 48.630220 48.63
